# Integrative neuromodulation in diabetic foot infections: electroacupuncture-driven macrophage reprogramming and synergy with antimicrobial biomaterials

**DOI:** 10.3389/fcimb.2026.1874221

**Published:** 2026-06-23

**Authors:** Yi Lu, Bingbing Gong, Taoyong Fu, Yujia Xie, Jiayu Huang, Wanyi Ma, Shu Wang, Yuyao Wu, Lilan Chen, Quanfu Chen

**Affiliations:** 1 The Affiliated Traditional Chinese Medicine Hospital, Guangzhou Medical University, Guangzhou, China; 2 Graduate School, Guangzhou Medical University, Guangzhou, China

**Keywords:** antimicrobial biomaterials, cholinergic anti-inflammatory pathway, diabetic foot infection, electroacupuncture, macrophage reprogramming, neuromodulation

## Abstract

Diabetic foot infections (DFIs) are characterized by persistent bacterial biofilms and impaired host immune defenses. Within the diabetic microenvironment, peripheral neuropathy disrupts neuro-immune feedback loops, contributing to the arrest of resident macrophages in a pro-inflammatory M1 phenotype. This phenotypic arrest impairs efferocytosis and sustains chronic inflammation, which conventional antimicrobial therapies frequently fail to resolve. Electroacupuncture (EA) serves as a neuromodulatory intervention capable of reversing this immune dysfunction. By stimulating specific somato-autonomic reflexes—including the vagal-adrenal and sympathoadrenal axes—EA activates the systemic cholinergic anti-inflammatory pathway (CAP). The subsequent release of neurotransmitters, acting on receptors such as the α7 nicotinic acetylcholine receptor (α7nAChR) on macrophages, inhibits pro-inflammatory cascades and promotes polarization toward the pro-repair M2 phenotype. Concurrently, EA-induced localized neuropeptide release and vasodilation mitigate the ischemic microenvironment, providing metabolic support for sustained pathogen clearance. Furthermore, this review proposes a translational “Vanguard and Commander” approach, integrating the localized biofilm-disrupting properties of advanced antimicrobial biomaterials (the “vanguard”) with the sustained immune reprogramming mediated by EA (the “commander”). This integration links material science with bioelectronic neuromodulation to coordinate the tissue repair process spatiotemporally. Ultimately, this combined approach provides an opioid-sparing, integrative therapeutic strategy for the management of refractory diabetic wound infections.

## 1 Introduction

Diabetic foot ulcers (DFUs) are a common and highly morbid complication of diabetes mellitus. Epidemiological data indicate that the lifetime risk for diabetic patients to develop these ulcers ranges from 19% to 34% (Jiang et al., 2023; McDermott et al., 2023). When these ulcers become infected DFIs, the risk of lower-extremity amputation rises sharply, and the five-year mortality rate can reach 50% to 70% (Jiang et al., 2023; McDermott et al., 2023). Current clinical management heavily relies on surgical debridement and systemic broad-spectrum antibiotics. However, these conventional strategies often fail to achieve complete healing (Davis et al., 2018; Burgess et al., 2021). This failure is largely attributed to the emergence of multidrug-resistant bacteria and the formation of complex, impenetrable biofilms within the wound bed (Fisher et al., 2017; Xiao et al., 2024a; Oliveira et al., 2025). Fundamentally, delayed healing and persistent infection are rooted in a compromised host immune response, where chronic inflammation fails to clear pathogens and blocks tissue repair (Wolf et al., 2021; Peng et al., 2025).

**Figure 1:**
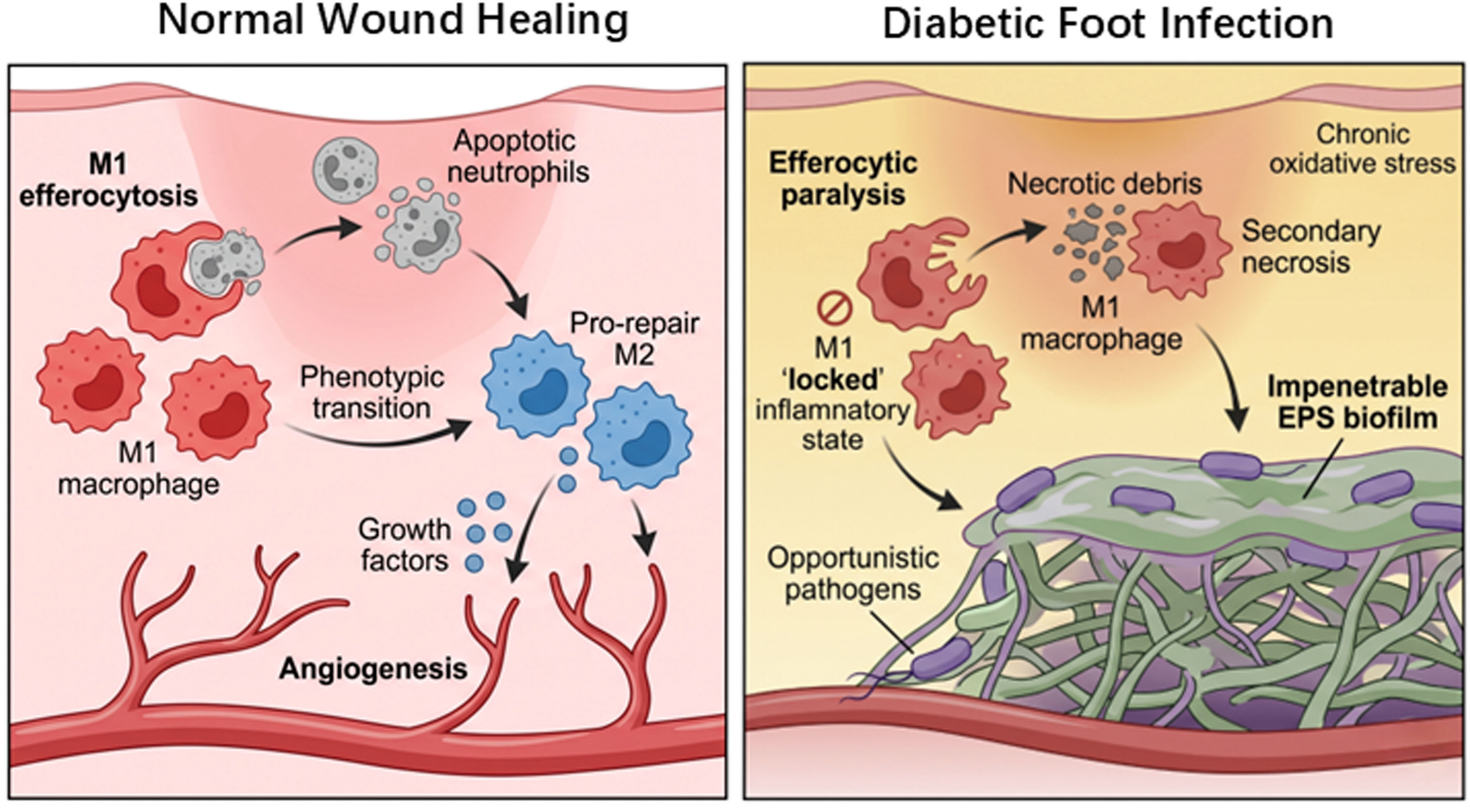
The immune crisis and structural fixation in diabetic foot infections (DFIs). In normal wound healing (left), effective efferocytosis of apoptotic cells triggers the transition of macrophages from a pro-inflammatory M1 state to a pro-repair M2 phenotype. Conversely, in the DFI microenvironment (right), chronic oxidative stress and metabolic dysfunction lead to efferocytic paralysis. Macrophages are locked in an M1 state, allowing opportunistic pathogens to construct impenetrable EPS biofilms. This vicious cycle perpetuates chronic inflammation and prevents tissue regeneration.

A growing body of evidence highlights macrophage dysfunction as a primary driver of this impaired immunity in diabetic wounds (Kloc et al., 2019; Louiselle et al., 2021; Sharifiaghdam et al., 2022; Blériot et al., 2023). Normally, macrophages exhibit dynamic plasticity, transitioning from a pro-inflammatory (M1) state to a pro-healing (M2) state to facilitate tissue regeneration (Wang et al., 2014; Sridharan et al., 2015). In the diabetic microenvironment, however, resident macrophages remain stalled in the M1 phenotype (**Figure 1**) (Rehak et al., 2022; Wu et al., 2022a; Cai et al., 2023). This persistent inflammatory state severely impairs their capacity for efferocytosis—the clearance of apoptotic cells—which is essential for initiating tissue repair (Clayton et al., 2024; Huang et al., 2025; Xie et al., 2025). Furthermore, this macrophage dysregulation is intimately connected to the loss of local neuro-immune cross-talk (Pavlov and Tracey, 2017). Diabetic peripheral neuropathy damages sensory nerves that normally release immunomodulatory neuropeptides, such as calcitonin gene-related peptide (CGRP), thereby severing a vital feedback loop required for macrophage polarization and proper wound healing (Kolter et al., 2019; Jiang et al., 2024; Peng et al., 2025).

**Figure 2:**
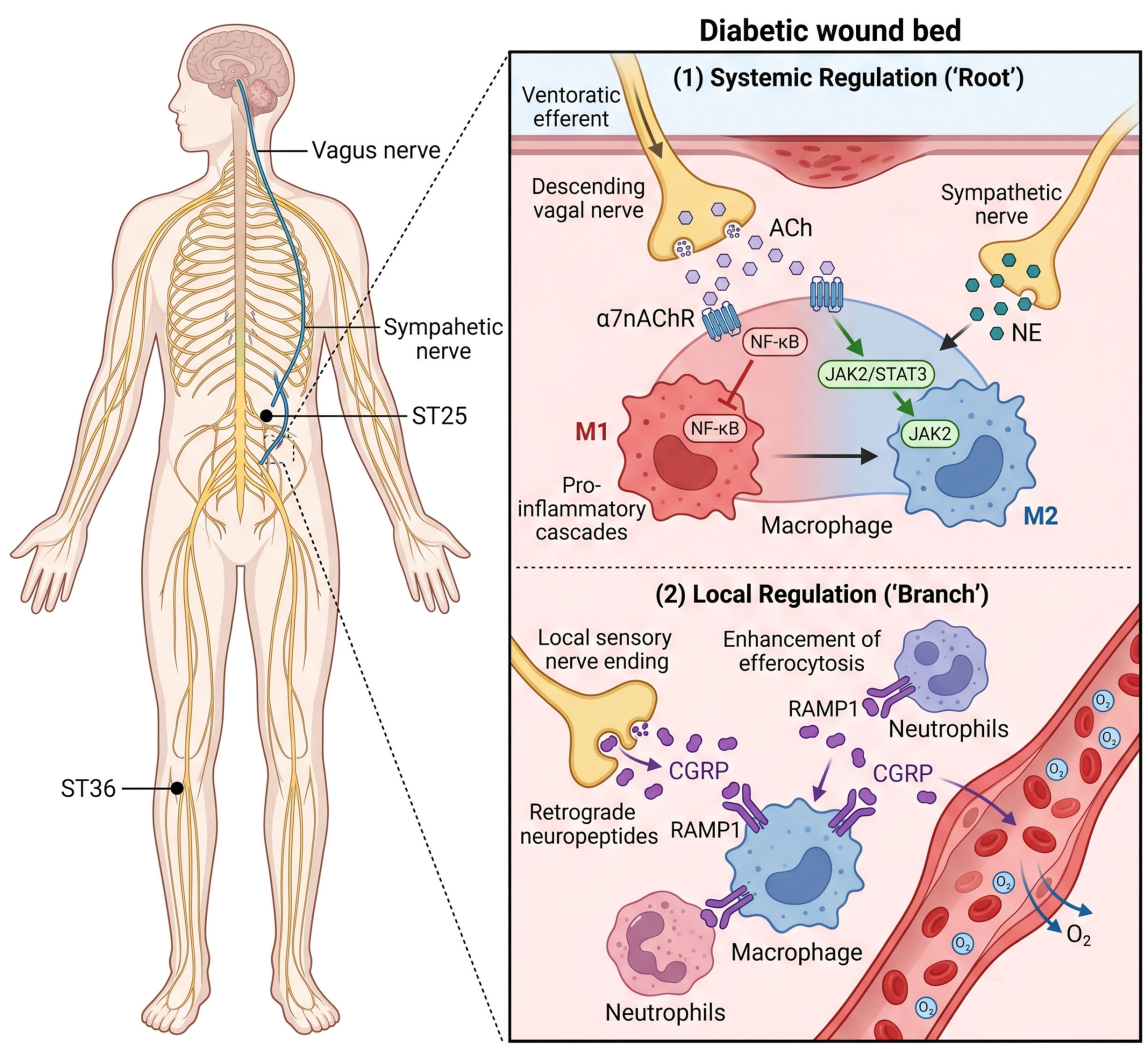
EA modulates macrophage polarization via hierarchical neuro-immune axes. EA at specific acupoints operates through the “Root and Branch” (Biao-Ben) theory. Systemically (Root), EA drives the vagal-adrenal axis to release ACh, which binds to α7nAChR on macrophages, inhibiting pro-inflammatory cascades. Locally (Branch), EA stimulates sensory nerve endings to release CGRP. CGRP acts on receptor activity-modifying protein 1 (RAMP1) receptors to enhance efferocytosis and induces neurogenic vasodilation, providing metabolic support for the pro-repair M2 phenotypic transition.

EA, derived from Traditional Chinese Medicine, provides a targeted approach to modulate both systemic and local neuro-immune networks (Li et al., 2021). EA utilizes the body’s innate somato-autonomic reflexes to regulate immune activity. Specifically, stimulation at acupoints like Zusanli (ST36) activates the vagal-adrenal axis and the CAP (Liu et al., 2021; Alen, 2022). This systemic reflex releases acetylcholine, which binds to the α7nAChR on macrophages to suppress excessive inflammation and promote M2 polarization (Keever et al., 2024; Liu et al., 2024). Concurrently, EA enhances local sympathetic and sensory nerve functions, promoting microvascular vasodilation and neuropeptide release to relieve tissue hypoxia (Zhang et al., 2023b; Xi et al., 2024). The hierarchical pathways mediating these effects are illustrated in **Figure 2**. Recognizing the limitations of standalone treatments, combining EA with advanced immunomodulatory and antimicrobial biomaterials offers a promising strategy (Davenport Huyer et al., 2020; Zhang et al., 2023a; Li et al., 2024a). Based on the traditional “Root and Branch” (Biao-Ben) concept, this review explores the mechanisms of EA in reversing macrophage paralysis and discusses the synergistic potential of integrating bioelectronic neuromodulation with intelligent biomaterials to treat refractory diabetic foot infections.

## 2 The immune crisis in DFI: macrophage dysfunction and biofilm persistence

Physiological wound healing is a tightly regulated process that depends heavily on the dynamic plasticity of innate immune cells. Among these, macrophages act as key orchestrators of tissue repair and homeostasis (Kloc et al., 2019; Mass et al., 2023). In a healthy immune response, macrophages initially adopt a pro-inflammatory (M1-like) phenotype to phagocytose invading pathogens and clear necrotic debris. Subsequently, specific cues in the microenvironment signal these cells to transition into an anti-inflammatory, pro-resolving (M2-like) phenotype, a shift that is essential for stimulating angiogenesis, extracellular matrix organization, and epithelialization (Aitcheson et al., 2021; Wculek et al., 2022). However, in the DFU microenvironment, this critical phenotypic switch is significantly disrupted. Chronic hyperglycemia, advanced glycation end-products (AGEs), and unrelenting oxidative stress act collectively to alter intracellular signaling and metabolic pathways (Jin et al., 2024). Recent studies also highlight the involvement of epigenetic modifications and specific signaling cascades, such as the stimulator of interferon genes (STING) pathway, in exacerbating this dysfunction (Geng et al., 2023; Song et al., 2025). Consequently, resident macrophages become “locked” in a persistent M1 state, resulting in a prolonged, self-sustaining inflammatory process that damages healthy tissue rather than repairing it (Wolf et al., 2021; Peng et al., 2025).

### 2.1 Phagocytic paralysis and the rise of biofilms

The complex pathological distinctions between normal wound healing and the DFI environment, including the macrophage dysfunction, efferocytic failure, and the presence of persistent biofilms, are consolidated in **Table 1**. A critical consequence of this polarization arrest is a substantial functional deficit: phagocytic and efferocytic paralysis. M1-locked macrophages in diabetic wounds exhibit a markedly reduced capacity to execute efferocytosis—the targeted clearance of apoptotic cells—which normally serves as a primary trigger for the M2 phenotypic switch (Aitcheson et al., 2021; He et al., 2024). The failure of efferocytosis leads to delayed clearance of apoptotic cells and the subsequent release of toxic intracellular contents via secondary necrosis, further exacerbating the hostile wound microenvironment (Huang et al., 2025; Xie et al., 2025).

**Table 1:** Pathological distinctions between normal wound healing and diabetic foot infection (DFI).

Feature	Normal wound healing	Diabetic foot infection (DFI)	References
Predominant Phenotype	Dynamic M1-M2 transition	Persistent M1-locked state	(Aitcheson et al., 2021; Wolf et al., 2021; Peng et al., 2025)
Phagocytic Activity	Effective pathogen clearance	Phagocytic dysfunction	(Aitcheson et al., 2021; Clayton et al., 2024)
Efferocytosis	Efficient clearance of apoptotic cells	Impaired efferocytosis; secondary necrosis	(Aitcheson et al., 2021; Huang et al., 2025; Xie et al., 2025)
Biofilm Status	Transient or absent	Persistent and structured biofilms	(Raziyeva et al., 2021; Xiao et al., 2024a; Oliveira et al., 2025)
Neuro-Immune Status	Intact feedback loops	Disrupted feedback; sensory neuropathy	(Jiang et al., 2024; Lu et al., 2024)
ROS & Oxidative Stress	Regulated physiological levels	Excessive accumulation; sustains chronic inflammation	(Aitcheson et al., 2021; Xiao et al., 2024a; Xu et al., 2026)

DFI, diabetic foot infection; DFU, diabetic foot ulcer; CGRP, calcitonin gene-related peptide; ROS, reactive oxygen species. These pathological imbalances significantly impair chronic wound healing.

Moreover, the inability of macrophages to efficiently process and recycle phagocytosed microbial cargo deprives them of essential immunometabolic fueling required for sustained antibacterial activity (Gonzalez et al., 2023; Lesbats et al., 2025). This metabolic failure contributes to an immunosuppressed, nutrient-rich niche. Opportunistic pathogens readily exploit this breakdown in immune surveillance, adhering to the wound bed and rapidly secreting extracellular polymeric substances (EPS) to form highly organized, impenetrable biofilms (Huang et al., 2025; Xie et al., 2025).

### 2.2 The structural fixation of the chronic wound

Once established, the EPS matrix of biofilms functions as a structural barrier that physically shields embedded bacteria from systemic antimicrobial agents and local immune cell infiltration (Fisher et al., 2017; Burgess et al., 2021). In addition to providing physical protection, biofilms release virulence factors that continuously stimulate pattern recognition receptors on adjacent immune cells. This interaction reinforces the M1 polarization state, thereby stalling the tissue repair process in an extended inflammatory phase (Raziyeva et al., 2021; Xiao et al., 2024a; Oliveira et al., 2025). Consequently, a self-sustaining loop develops: impaired immune clearance allows for biofilm maturation, while the mature biofilm perpetuates non-resolving inflammation (Cai et al., 2023). Overcoming this combined structural and immunological barrier requires therapeutic strategies capable of modulating local inflammatory signaling and guiding macrophage phenotypes toward a reparative state (Davis et al., 2018; Clayton et al., 2024).

## 3 EA modulates macrophage polarization: the neuro-immune axis

EA translates mechanical and electrical stimuli at specific acupoints into neural signals that regulate immune homeostasis (Li et al., 2021). Electrical stimulation, a core component of EA, has been shown to directly influence macrophage behavior, promoting a shift from the M1 to the M2 phenotype and modulating local inflammatory transcriptional networks (Cheah et al., 2021; Bianconi et al., 2024). Grounded in the traditional “Root and Branch” (*Biao-Ben*) approach, the therapeutic efficacy of EA in DFIs relies on activating systemic neuro-immune circuits while improving the local wound microenvironment (Xi et al., 2024). Addressing the persistent microvascular impairment and neuro-immune decoupling in the diabetic niche is a primary target of these neuromodulatory interventions (Ge et al., 2025).

### 3.1 Systemic reprogramming: the neuro-reflexive macrophage modulation (treating the “root”)

The distinct systemic neuro-immune pathways activated by EA are consolidated in **Table 2**. The systemic modulation of macrophage polarization involves the activation of somato-autonomic reflexes mapped to specific neuroanatomical substrates. Research indicates that low-intensity EA at hindlimb acupoints, such as ST36, drives the vagal-adrenal axis by stimulating a specific population of prokineticin receptor 2 (PROKR2)-expressing sensory neurons that project to the hindbrain (Liu et al., 2021). This reflex activates the systemic CAP, a neural mechanism regulating the inflammatory response (Li et al., 2021; Alen, 2022). Efferent vagal signals trigger the release of acetylcholine (ACh), which binds to the α7nAChR expressed on macrophages (Keever et al., 2024; Liu et al., 2024). Studies utilizing genetic knockout models demonstrate that the anti-inflammatory effects of EA are abolished in α7nAChR-deficient mice, indicating the receptor’s role in suppressing pro-inflammatory cascades and promoting M2-associated Janus kinase 2 (JAK2)/signal transducer and activator of transcription 3 (STAT3) signaling (Liu et al., 2024).

**Table 2:** Hierarchical neuro-immune regulatory pathways of electroacupuncture (EA) in the management of DFI.

Regulatory level	Acupoint	Neural pathway	Mediators/receptors	Immunomodulatory effect	References
Systemic ("Root")	ST36	PROKR2+ sensory neurons → Vagal-adrenal axis	ACh → α7nAChR	Suppression of systemic TNF-α, IL-6, and neuroinflammation; JAK2/STAT3 activation	(Li et al., 2021; Liu et al., 2021; Keever et al., 2024)
Systemic ("Root")	ST25	Sympathoadrenal medullary axis	NE	Upregulation of IL-10; Downregulation of pro-inflammatory cytokines	(Zhang et al., 2023b; Xi et al., 2024)
Local ("Branch")	Local sites	Retrograde sensory nerve activation	CGRP → RAMP1	Promotion of efferocytosis; M2 polarization; Microvascular vasodilation	(Jiang et al., 2024; Lu et al., 2024)
Metabolic Support	Systemic/Local	Autonomic microcirculatory regulation	Increased O_2_ & Nutrient supply	Supports OXPHOS-dependent M2 phagocytic activity	(Wculek et al., 2022; Gonzalez et al., 2023; Ge et al., 2025)

EA, electroacupuncture; ACh, acetylcholine; α7nAChR, alpha7 nicotinic acetylcholine receptor; NE, norepinephrine; CGRP, calcitonin gene-related peptide; RAMP1, receptor activity-modifying protein 1; OXPHOS, oxidative phosphorylation. The spatiotemporal coordination of these pathways modulates the inflammatory microenvironment in DFIs.

Furthermore, EA recruits the sympathoadrenal medullary axis to modulate regional immunity. Stimulation at abdominal acupoints like Tianshu (ST25) elevates the firing rate of the adrenal sympathetic nerve, increasing peripheral norepinephrine (NE) levels (Zhang et al., 2023b). This neural activation exerts anti-inflammatory effects by downregulating pro-inflammatory cytokines such as IL-6 and IL-1β, while concurrently upregulating the anti-inflammatory cytokine IL-10 (Zhang et al., 2023b). These systemic pathways represent the “Root” (*Ben*) treatment, functioning to reverse systemic immune dysregulation and restore host-level metabolic and immunological homeostasis (Li et al., 2021; Wculek et al., 2022; Ge et al., 2025).

### 3.2 Local microenvironmental rescue: neuropeptides and vasodilation (treating the “branch”)

While systemic reflexes modulate general host immunity, EA applied at local “Branch” (*Biao*) sites targets the regional wound microenvironment. Diabetic wounds often present with endothelial dysfunction and inflammatory microvascular remodeling, conditions that impair normal tissue repair (Ge et al., 2025). The application of EA stimulates local sensory nerves, particularly nociceptive fibers, inducing the retrograde release of neuropeptides such as CGRP (Jiang et al., 2024; Lu et al., 2024). Research indicates that CGRP acts via the receptor activity-modifying protein 1 (RAMP1) receptor on local immune cells to inhibit excessive neutrophil recruitment, promote neutrophil apoptosis, and enhance macrophage efferocytosis (Ren et al., 2023; Lu et al., 2024).

This CGRP-mediated signaling, which partly involves thrombospondin-1 (TSP-1) release, guides macrophage polarization toward the M2 phenotype (Lu et al., 2024). The restoration of these neuro-immune interactions in diabetic models with peripheral neuropathy has demonstrated accelerated wound closure (Bosch-Queralt et al., 2023; Lu et al., 2024). Furthermore, local neurogenic vasodilation helps alleviate tissue hypoxia and ischemia, supplying the necessary metabolic substrates for M2 macrophages to maintain pathogen clearance and support tissue regeneration (Lesbats et al., 2025). Upon engulfing pathogens, macrophages can recycle bacterial cargo into various metabolic pathways to fulfill bioenergetic demands, a process associated with increased oxidative phosphorylation (OXPHOS) and mitochondrial content (Wculek et al., 2022; Gonzalez et al., 2023; Lesbats et al., 2025). Through the coordination of these systemic and local neuro-immune networks, EA modulates the DFI microenvironment to facilitate tissue repair. The specific hierarchical pathways involved in this regulation are summarized in **Table 2**.

## 4 Translational synergy: integrating EA with antimicrobial biomaterials

**Figure 3:**
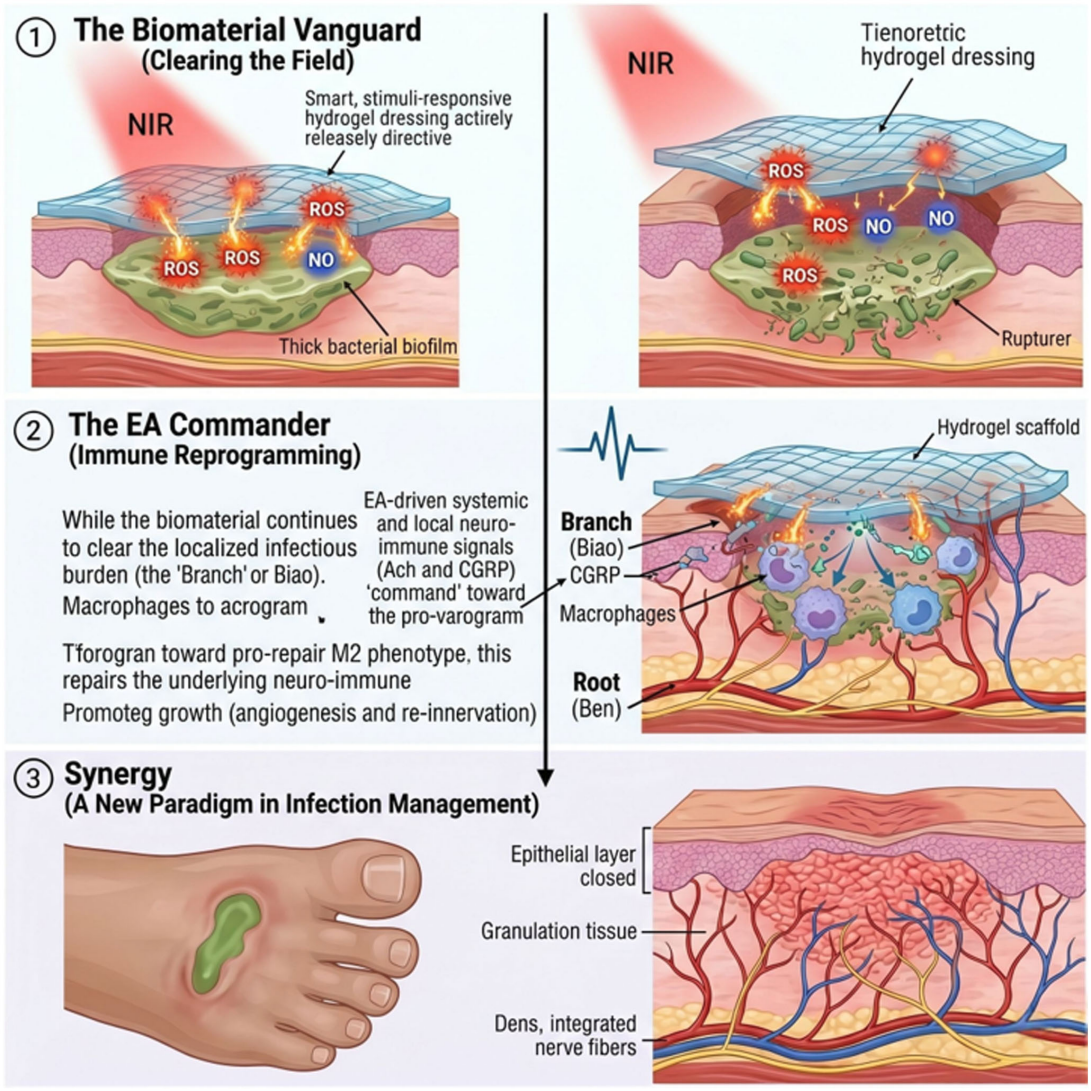
The “Vanguard and Commander” translational synergy in DFI management. Integrating advanced antimicrobial biomaterials with EA achieves a profound spatiotemporal synergistic effect. (1) The Biomaterial Vanguard: Stimuli-responsive hydrogels and nanozymes rapidly dismantle recalcitrant biofilms via precise ROS/NO release, acutely reducing the bacterial burden. (2) The EA Commander: Concurrently, EA provides sustained systemic and local neuromodulation, reprogramming macrophages toward a pro-repair M2 state and enhancing local perfusion. (3) Synergy: This combined bioelectronic and biomaterial approach successfully breaks the immune paralysis, accelerating robust vascularized tissue regeneration. The human-body and foot elements in **Figure 2** and **3** are schematic illustrations and do not contain actual photographs or identifiable images of human subjects or patients.

While neuromodulation via EA provides a mechanism for reversing immune dysfunction, severe, multidrug-resistant infections necessitate immediate pathogen reduction (Sun et al., 2024). Traditional therapeutic approaches face limitations: neuromodulation requires time to rebuild the host’s immunological microenvironment, whereas standalone antimicrobial biomaterials often fail to achieve complete tissue repair because the underlying host immune impairment remains unaddressed once the active agents are depleted (Cai et al., 2023; Huang et al., 2023). Recurrent infections and non-healing wounds significantly affect patient survival, necessitating integrated translational strategies (Jiang et al., 2023; Jia et al., 2025). This synergistic approach represents a “Vanguard and Commander” therapeutic model (**Figure 3**).

### 4.1 The “Vanguard”: rapid pathogen knockdown via advanced biomaterials

Recent advancements in materials science have introduced engineered systems that serve as the “vanguard” in the wound bed. Nanozyme-based therapeutic systems, characterized by stable catalytic activity and tissue permeability, can integrate multifaceted interventions such as antibacterial action, glycemic control, and oxygen delivery (Pu et al., 2024; Xiao et al., 2024a; Jia et al., 2025). Sophisticated biomaterials, including stimuli-responsive hydrogels and microneedle platforms, utilize specific crosslinking strategies to achieve dynamic responsiveness to the pH, glucose, and ROS levels of the wound microenvironment (Wu et al., 2022b; Liu et al., 2025; Zhang et al., 2025a; Xu et al., 2026).

These systems physically disrupt bacterial biofilms through multimodal strategies, including near-infrared (NIR) photothermal therapy (PTT), chemodynamic therapy (CDT), ultrasound-triggered mechanisms, and the controlled release of antimicrobial gases like nitric oxide (NO) and hydrogen sulfide (H_2_S) (Hou et al., 2024; Huang et al., 2024; Chen et al., 2025; Li et al., 2025; Shen et al., 2025). By generating ROS or peroxynitrite (ONOO^-^) *in situ*, or conversely scavenging excessive ROS to remodel the microenvironment and delivering essential growth factors, these materials rapidly reduce the localized infectious burden (Nurkesh et al., 2020; Guo et al., 2024; Yuan et al., 2025). However, the exhaustion of these passive clearing agents often leaves residual “persister” bacteria, highlighting the necessity for a sustained endogenous immune response to prevent reinfection (Fisher et al., 2017; Yu et al., 2024).

### 4.2 The “commander”: sustained immune reprogramming via EA

In this synergistic model, EA functions as the “commander” by systemically and locally modulating the innate immune response. While the biomaterial addresses the immediate microbial load, EA sustains the activation of α7nAChR on wound-resident macrophages through somato-autonomic reflexes, supporting the transition to the M2 phenotype (Li et al., 2021; Liu et al., 2024). This neural and hormonal regulation aids in the clearance of bacterial debris and necrotic tissue dispersed by the biomaterial (Lu et al., 2024; Ge et al., 2025).

Furthermore, the neuromodulatory function of EA addresses vascular impairments that topical materials may not fully cover. By modulating cardiac and adrenal sympathetic activities, EA regulates systemic immune-inflammatory homeostasis and regional microcirculatory perfusion (Zhang et al., 2023b; Xi et al., 2024). The resulting localized neurogenic vasodilation, mediated by neuropeptides such as CGRP, supplies oxygen and nutrients to support M2 macrophage metabolism and enhances the tissue distribution of active agents released by the hydrogel scaffolds (Lu et al., 2024; Ge et al., 2025). Additionally, combinations of these approaches can incorporate localized delivery systems, such as exosomes or targeted nanoparticles, to further augment vascular regeneration alongside neuromodulation (Li et al., 2024b; Yang et al., 2024).

### 4.3 Achieving “1 + 1>2”: a new system in infection management

The integration of bioelectronic stimulation and biomaterials creates a synergistic effect by spatiotemporally coordinating the tissue repair process (Sridharan et al., 2015). Temporal immunomodulatory hydrogels can mimic neuro-immune-vascular cascades, utilizing early-stage agent release to inhibit infection and sustained release to promote M2 polarization and angiogenesis (Yu et al., 2021; Wang et al., 2026). Recent developments in responsive hydrogels and nanocomposites further support this immunoregulation, allowing for precise control over the wound metabolic microenvironment (Yu et al., 2021; Gong et al., 2025; Tan et al., 2025).

This combined application links material science with neuromodulation. The biomaterial dismantles the immediate microbial threat (addressing the “Branch” or *Biao*), while EA repairs the underlying neuro-immune communication network (addressing the “Root” or *Ben*). By optimizing endogenous macrophage function and reducing reliance on systemic broad-spectrum antibiotics, this integrated strategy presents a targeted, opioid-sparing therapeutic option for the management of refractory diabetic wound infections (Jiang et al., 2023; Soliman et al., 2025; Zhang et al., 2025a).

## 5 Conclusion

The persistent challenge of DFUs is fundamentally rooted in host immune impairment. Chronic metabolic stress and neurological deficits maintain resident macrophages in a dysfunctional, pro-inflammatory state, creating an immunosuppressed niche that facilitates the structural fixation of recalcitrant bacterial biofilms (Eming et al., 2017; Aitcheson et al., 2021; Raziyeva et al., 2021; Clayton et al., 2024). These clinical issues significantly affect patient outcomes, highlighting the need for integrative therapeutic strategies that extend beyond topical bacterial eradication (Jiang et al., 2023).

EA serves as an evidence-based neuromodulatory intervention that modulates this localized dysfunction. By activating distinct somato-autonomic reflexes—such as the CAP and the sympathoadrenal medullary axis—EA restores systemic immune homeostasis (Chavan et al., 2017; Li et al., 2021; Zhang et al., 2023b; Liu et al., 2024). Concurrently, EA-induced local vasodilation, mediated by the release of neuropeptides like CGRP, mitigates the hypoxic microenvironment and provides the metabolic foundation for sustained macrophage phagocytosis and the transition toward a pro-repair M2 state (Lu et al., 2024; Lesbats et al., 2025). This dual regulation reflects the neuro-immunological translation of the “Root and Branch” (*Biao-Ben*) approach (Xi et al., 2024).

## 6 Clinical feasibility, advantages, and limitations

To bridge the gap between molecular mechanisms and macroscopic clinical outcomes, it is essential to recognize how cellular immuno-stimulation improves patient conditions. Clinically, the EA-driven shift toward M2 macrophages and the restoration of local vasodilation directly translate to accelerated granulation tissue formation, reduced necrotic wound exudate, alleviation of neuropathic pain, and a decreased overall risk of lower-extremity amputation. While other approaches to rebuilding the immunological microenvironment exist—such as the topical application of recombinant growth factors, exogenous cytokines, or mesenchymal stem cell (MSC) therapies—EA offers distinct clinical advantages. As an opioid-sparing and non-pharmacological modality, EA safely leverages the body’s endogenous somato-autonomic reflexes, circumventing the systemic toxicity, high costs, and unstable delivery challenges frequently associated with exogenous immunomodulators.

Although clinical trials specifically combining EA with advanced stimuli-responsive nanomaterials for DFIs are currently traversing the preclinical pipeline, the foundational *in vivo* evidence supporting their spatiotemporal “1 + 1>2” synergy is highly promising. Nevertheless, the clinical application of EA in DFI management is not without limitations. Its feasibility is currently hindered by a lack of globally standardized treatment dosimetry—including optimal stimulation frequencies, duration, and acupoint selection protocols. Furthermore, the efficacy of EA is inherently operator-dependent and requires sustained patient compliance over multiple sessions. Most importantly, while EA serves as a powerful neuromodulatory adjunct to enhance tissue viability and immune defense, it cannot substitute for immediate surgical debridement, revascularization, or systemic antibiotic therapy in patients presenting with severe, end-stage gangrene or systemic sepsis.

## 7 Future perspectives

Moving forward, several critical areas warrant further investigation to optimize DFI management. First, understanding the epigenetic regulatory mechanisms—such as DNA methylation and noncoding RNAs—that govern macrophage plasticity in the diabetic niche will provide novel molecular targets for reversing M1-phenotype arrest (Wolf et al., 2021; Song et al., 2025). Additionally, exploring the cellular crosstalk between macrophages and other wound-resident cells, such as healing-associated fibroblasts, or utilizing mesenchymal stem cell-derived exosomes for targeted cellular reprogramming, presents a promising frontier in regenerative immunology (Li et al., 2024b; Xiao et al., 2024b).

Furthermore, as indicated by recent bibliometric analyses, the development of functionalized biomaterials remains a central research hotspot (Zhang et al., 2025b). Future studies should focus on optimizing the spatiotemporal delivery of immunomodulators, integrating advanced nanocomposites, dynamic microenvironment-modulating dressings, or piezoelectric hydrogels with bioelectronic stimulation to maximize neurovascular regeneration (Cheng et al., 2024; Hou et al., 2024; Wang et al., 2025).

In conclusion, utilizing integrative neuromodulation to restore macrophage function, particularly when combined with advanced stimuli-responsive biomaterials, offers a targeted and sustainable pathway to manage refractory DFIs.
